# Male partner involvement in postnatal care service utilization and its associated factors in Wolaita Sodo, southern Ethiopia, 2023

**DOI:** 10.3389/fgwh.2025.1481164

**Published:** 2025-02-18

**Authors:** Aklilu Adisu, Wubshet Estifanos, Genet Asefa, Fikre Moga Lencha, Addisalem Haile, Fekadu Abera Kebede

**Affiliations:** ^1^ College of Health Sciences and Medicine, Wolaita Sodo University, Sodo, Ethiopia; ^2^ College of Medicine and Health Sciences, Arba Minch University, Arba Minch, Ethiopia; ^3^ College of Health Sciences, Oda Bultum University, Chiro, Ethiopia

**Keywords:** male partner, postnatal service, involvement, Wolaita Sodo, Ethiopia

## Abstract

**Background:**

Involvement of male partners in postnatal care (PNC) is an effective approach to improving maternal and child health outcomes. Despite this, it has been perceived as a woman's responsibility and continues to be a significant problem in developing countries, including Ethiopia. Furthermore, there is a paucity of evidence regarding male involvement during postnatal care in Ethiopia, particularly in the study area. Therefore, the purpose of this study was to assess the current status of male partners’ involvement in postnatal care and associated factors in the study area, Wolaita Sodo, in southern Ethiopia in 2023.

**Methods:**

A community-based cross-sectional study design was conducted on 629 participants from 1 April to 1 May 2023 using a multistage sampling technique. A pre-tested and structured questionnaire was used to collect data. Data were entered into EpiData version 4.6 and exported to Statistical Package for Social Science (SPSS) version 25 for analysis. Bivariable and multivariable logistic regression analyses were employed to identify factors associated with male partner involvement during postnatal care. The level of significant association in the multivariable analysis was determined based on a *P*-value of <0.05.

**Results:**

This study found that 32.95% [95% confidence interval (CI): 29.2–36.8] of male partners are involved in postnatal care service utilization. A higher maternal educational level [adjusted odds ratio (AOR): 2.95, 95% CI: 1.76–4.94], good knowledge of postnatal care services (AOR: 3.2, 95% CI: 1.93–5.3), good knowledge of danger signs and complications (AOR: 4.5, 95% CI: 2.39–8.48), a favorable attitude (AOR: 4.02, 95% CI: 2.50–6.45), distance (AOR: 1.91, 95% CI: 1.15–3.17), and cesarean delivery (AOR: 2.5, 95% CI: 1.39–4.60) were significantly associated with male partner involvement in postnatal care services.

**Conclusion:**

In this study, a male partner's involvement in their spouse's PNC service utilization was found to be low. Maternal educational status, good knowledge of PNC services, good knowledge of danger signs and complications, a favorable attitude, distance, and cesarean delivery were associated with male partner’s involvement in PNC. Therefore, strengthening awareness about postnatal care services and danger signs through health education and promoting a positive attitude toward postnatal services are essential.

## 1 Introduction

Men and women are often assigned different roles and responsibilities by society, which significantly influence their health-seeking behaviors, including maternal healthcare services (1). In many cultures, maternal and child health (MCH) is perceived as primarily the responsibility of women, which can limit male partner involvement in crucial aspects of maternal care, such as postnatal care (PNC). Male partner involvement in MCH is defined as actively supporting and promoting the health and wellbeing of their partners and children (2).

The World Health Organization (WHO) advocates for male participation in pregnancy, labor, and the postpartum period as a practical approach to improving health outcomes for both mothers and newborns (3). Evidence has shown that male involvement in maternal health programs positively influences maternal, neonatal, and child health (4).

Despite the benefits of male engagement, male participation in PNC remains critically low in many developing countries, including Ethiopia, where maternal and neonatal mortality rates remain high (5). Poor male involvement in postnatal healthcare contributes to an increase in maternal and newborn morbidity and mortality in multiple ways (6).

In 2017, a total of 295,000 women worldwide died as a result of complications during and after pregnancy and childbirth. Regions with limited resources, such as southern Asia and sub-Saharan Africa (SSA), accounted for more than 254,000 of these maternal deaths, as indicated by the estimated global maternal mortality rate (7). According to the Ethiopian Demographic and Health Survey 2019, the country's maternal mortality ratio was projected to be at 401 deaths per 100,000 live births, while the neonatal mortality rate stands at 33 deaths per 1,000 live births (8). These figures underscore the urgent need for increased male involvement in postnatal care to improve maternal and child health outcomes. However, male engagement in PNC is often minimal (5, 9).

Despite the WHO's recommendation and effort to promote male partner involvement in maternal and child healthcare in low- and middle-income countries (LMICs), these countries, including Ethiopia, place less emphasis on male partners’ involvement in maternity and childcare, particularly PNC (10).

Research conducted in Nepal and Nigeria revealed that male involvement in postnatal care visits was only 33.8% and 20%, respectively (11, 12). In Ethiopia, studies conducted in Addis Ababa (13), Sidama Zone (9), and Motta District (14) indicated that 54.7%, 11.8%, and 20.8% of male partners utilized postnatal care alongside their spouses, respectively. These findings highlight the need for further research to understand the magnitude and underlying factors affecting male involvement in postnatal care and to identify strategies to increase their participation.

The limited engagement of men in postnatal care is primarily attributed to the belief that maternal health is solely a woman's concern and the potential lack of awareness among men regarding the importance of their involvement in postnatal care services (15). In addition, cultural and social norms may discourage male participation in postnatal care services (16). Furthermore, various factors such as socio-demographic, individual, cultural, and health facility-related aspects make it challenging for male partners to participate in postnatal care services, as indicated by previous studies (1, 17).

Ethiopia has made substantial efforts to enhance male partner engagement through the health extension program and the health development army. Nevertheless, the issue still persists as a public health concern. To tackle this issue, the Federal Ministry of Health underscored the necessity for further research and interventions in the country (18).

Existing studies on postnatal care have primarily focused on women's perspectives, with limited attention given to male partner involvement. In addition, most research on male partners has relied on a 12-month recall period, which can lead to recall bias and inaccurate reporting of involvement. This study addresses these gaps by specifically examining male partner involvement within the first 6 months post-birth, a period critical for maternal and infant health. The 6-month window minimizes recall bias and ensures more accurate data, while also providing insights into the factors influencing male involvement in postnatal care in Wolaita Sodo, southern Ethiopia. This research contributes to a better understanding of male partner roles in postnatal care, offering valuable insights for improving health programs and policies in the region.

## 2 Methods and materials

### 2.1 Study area and period

This study was conducted in Sodo, Wolaita Zone. Wolaita Sodo is found in the Wolaita Zone in the Southern Nations, Nationalities, and People’s Region (SNNPR) of Ethiopia and is located 346 km from Addis Ababa, the capital city of Ethiopia. Sodo is divided into seven administrative sub-cities, which are further subdivided into 25 kebeles, the smallest administrative units in Ethiopia. According to the Sodo health office, the total population is estimated to be 250,312, of which 122,653 are men. The total number of mothers who gave birth in the last 6 months was 2,917. The city has one comprehensive specialized university hospital, 3 private hospitals, 3 health centers, 14 health posts, and 34 private health institutions (19). This study was conducted from 1 April to 1 May 2023.

### 2.2 Study design and population

A community-based cross-sectional study was conducted. The source population included all male partners whose wives gave birth in the last 6 months in Wolaita Sodo. The study population consisted of all male partners whose wives gave birth in the last 6 months in selected kebeles in Wolaita Sodo during the data collection period. The inclusion criteria were all male partners whose wives gave birth in the last 6 months in selected Kebeles and who lived in the study area for at least 6 months. The exclusion criteria excluded male partners who were critically ill or had communication difficulties, and mothers who gave birth outside of wedlock.

### 2.3 Sample size determination

The sample size of the study was determined by using the single population proportion formula based on the assumption of a 95% confidence interval (CI), a margin of error (d) of 5%, a 10% non-response rate, a design effect of 1.5, and the proportion of male partner involvement (54.7%) taken from a study conducted in Addis Ababa (14). Thus, the sample size was found to be 629.

### 2.4 Sampling technique and procedure

A multistage sampling technique was used to select a representative sample. Initially, from the 25 kebeles found in the city, 8 kebeles were selected randomly. The list of households with mothers who had given birth in the past 6 months was obtained from the health extension workers’ registry. However, the precise location of the households was not always clear from the registry alone as it only provided a list of mothers without specific addresses. To overcome this challenge, we worked closely with the health extension workers to identify and locate the households. In some cases, the health extension workers accompanied the research team to guide us to the correct locations.

Once the households were located, the sample size was proportionally allocated to each selected kebele. Male partners whose spouses gave birth in the last 6 months before the study were selected using systematic random sampling. The starting point for the selection was determined through a lottery method, and subsequent households were selected at a fixed interval (every second household) until the required sample size was reached. In cases where households were unavailable during the first visit, we revisited these households up to three times. If a household remained absent after multiple visits, it was replaced with the next eligible household on the list to maintain the sampling process.

### 2.5 Data collection instruments and procedures

The data were collected using a pre-tested structured interviewer-administered questionnaire adapted from different related studies in the literature (14). The questionnaire contains items on socio-demographic characteristics, knowledge about PNC services and danger sign during the PNC period, attitude toward PNC services, and health facility and involvement-related items. Male involvement is a composite variable without a single standard measurement scale. It was assessed using a 10-point index with dichotomous (yes/no) answers (14). Four diploma midwives were recruited for data collection. Two BSc midwives were also recruited for supervision of the data collection process. One day of training was given on how to collect the data, approach the study participants, interview techniques, and how to store the information. Before the actual data collection, a pretesting of the data collection tool was conducted. For households with no eligible male partner, the immediate next household was selected and then subsequent households were selected according to the already predetermined order. In cases of absence, households were visited three times to reduce non-participation.

### 2.6 Data quality control

To ascertain the quality of the data, a pretest was conducted on 5% of the calculated sample size outside of the study area (Areka). Thereafter, amendments were made accordingly. One day of training was given to data collectors and supervisors regarding the objective of the study, the data collection tool, and ways of data collection. Furthermore, close supervision was ensured on each data collection day.

### 2.7 Operational definitions

**Male partner**: A male partner was defined as a man who married a woman and was responsible for the pregnancy of that woman (14).

**Male partner involvement**: Male partner involvement was measured by considering 10 components. Each of these 10 elements was given a score of (1) when the participant performed the activity and (0) when the activity was not performed. A total score was calculated and 50% was used as a cut-off point to classify participants as either involved (those who scored 50% or above in the total score) or not involved (those who scored less than 50% in the total score) (14).

**Accompanying male partner to PNC service**: A male partner who went to the PNC clinic with his spouse for at least one PNC visit but not including the first 6-h PNC visit (14).

**Knowledge about PNC services**: Five knowledge-related items were used to assess the male partner's knowledge of PNC services. Each of these questions was given a score of (1) when answered correctly and (0) when the answer was incorrect. A total score was computed for each participant and the mean score was computed (14).

**Good knowledge about PNC services**: A male partner was deemed to have good knowledge about PNC services if they scored the mean score or above.

**Good knowledge of danger signs**: A male partner was deemed to have good knowledge of danger signs when they named three or more maternal danger signs or complications (14).

**Poor knowledge of danger signs**: A male partner was deemed to have poor knowledge of danger signs when they named replied fewer than three maternal danger signs or complications.

**Attitude**: The participants were asked seven attitude-related questions concerning PNC service utilization. The questions were designed using a Likert scale format with five answer alternatives ranging from strongly agree, agree, neutral, disagree, and strongly disagree, and a mean score was computed (14).

**Favorable attitude toward PNC services**: A male partner was deemed to have a favorable attitude toward PNC services if they scored the mean score or above.

### 2.8 Data processing and analysis

After the data were collected, it was checked for completeness and coded before being entered. Then, the data were entered into EpiData version 4.6 statistical software and then exported to Statistical Package for Social Science (SPSS) version 25 for analysis. Descriptive statistics including percentages, frequency, mean, standard deviation, tables, and graphs were used to present the characteristics of the study participants. The binary logistic regression model was fitted to identify risk factors for male partner involvement during PNC. Initially, a bivariable analysis was conducted to identify the candidate explanatory variables for the multivariable analysis. Thereafter, all explanatory variables with a *P*-value of less than 0.25 in the bivariable analysis were included in the multivariable logistic regression analysis to control for the effect of possible confounders and identify independent predictors of male partners’ involvement in the final model. Model fitness for the final model was checked using the Hosmer–Lemeshow goodness of fit test (0.346). Multicollinearity was checked using the variance inflation factor (VIF < 10). Adjusted odds ratios (AORs) with 95% CI and a *P*-value of less than 0.05 were used to determine the level of significance.

## 3 Results

### 3.1 Socio-demographic characteristics of the male partners and their families

In this study, a total of 610 participants were interviewed resulting in a response rate of 96.9%. The mean age was 33.24 years (± 4.961 SD) and 351 (57.6%) fell within the age group of 25 to 35 years. In terms of religious affiliation, 299 respondents (49.0%) identified as Protestant. In addition, 200 participants (32.8%) had received a primary education, 226 (37.0%) were engaged in merchant occupations, and 328 (53.8%) had three or more children (Table 1).

**Table 1:** Socio-demographic characteristics of male partners and their families in Wolaita Sodo, southern Ethiopia, 2023 (*N* = 610).

Variable	Category	Frequency	Percentage
Age (years)	18–25	55	9.0
	26–35	351	57.6
	>35	204	33.4
Religion	Orthodox	264	43.3
	Protestant	299	49.0
	Muslim	30	4.9
	Other^a^	17	2.8
Male partners’ education	No formal education	142	23.3
	Primary (1–8)	200	32.8
	Secondary (9–12)	132	21.6
	College or above	136	22.3
Wives’ education	No formal education	131	21.5
	Primary (1–8)	216	35.4
	Secondary (9–12)	111	18.2
	College or above	152	24.9
Male partners’ occupation	Merchant	226	37.0
	Government employed	128	21.0
	Self-employed	221	36.3
	Daily laborer	29	4.7
	Student	6	0.9
Wives’ occupation	Housewife	202	33.1
	Merchant	132	21.6
	Government employed	104	17.0
	Self-employed	130	21.3
	Student	42	6.9
Number of children	1–2	282	46.2
	≥3	328	53.8
Average monthly income (ETB)	<4,500	260	42.6
	≥4,500	350	57.4

ETB, Ethiopian birr.

^a^ Others: Catholic or Adventist.

### 3.2 Male partners’ knowledge of postnatal care services

**Figure 1:**
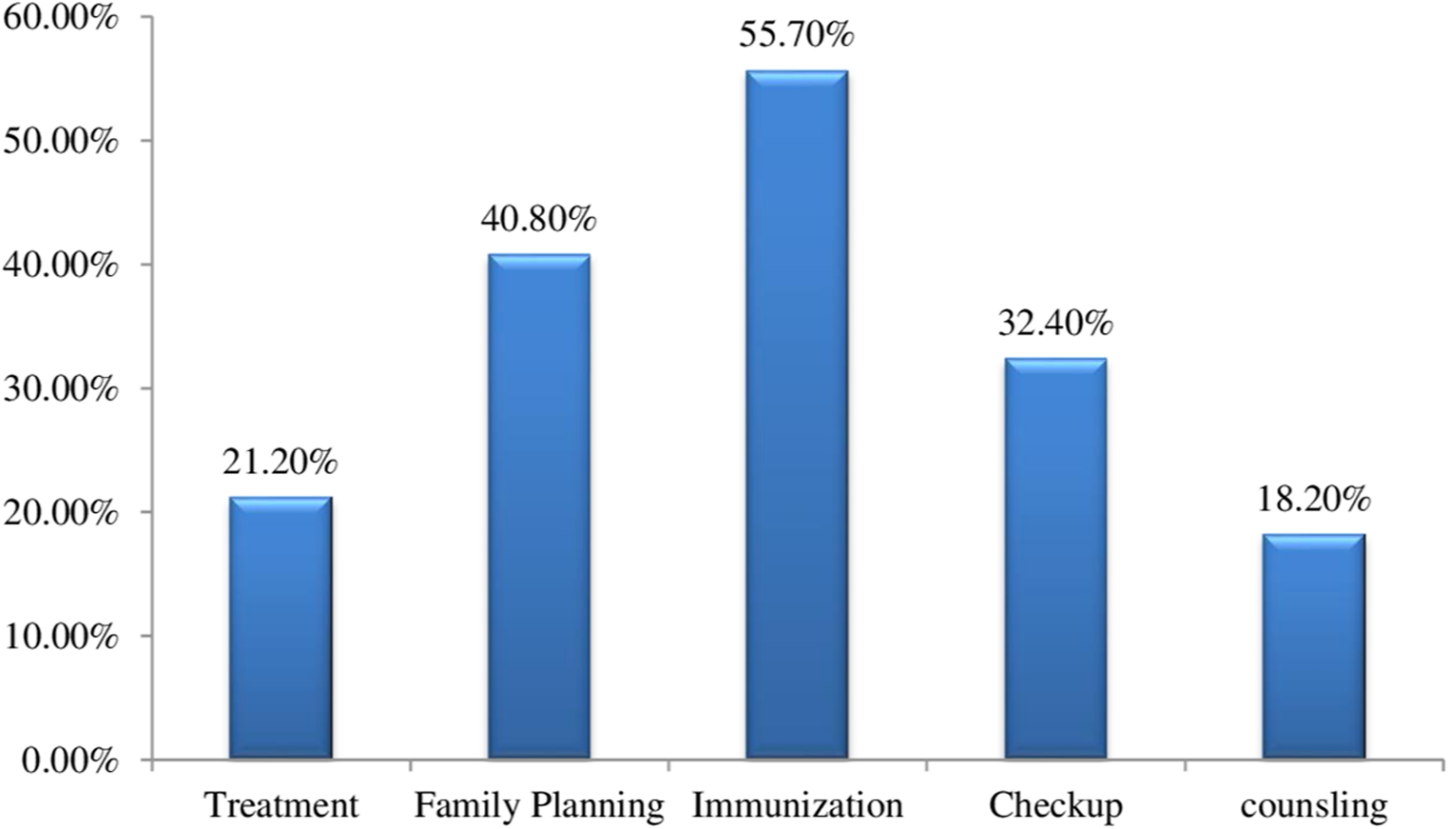
Male partner's knowledge of PNC services in Wolaita Sodo, southern Ethiopia, 2023 (*N* = 610).

From the total, 44.6% of male participants demonstrated good knowledge of PNC services. Among these respondents, 102 (16.7%) knew of the four PNC visits and 216 (35.4%) respondents mentioned the sixth-week PNC visit. In addition, 340 respondents (55.7%) identified immunization as one of the services provided during PNC visits (Figure 1).

### 3.3 Male partners’ knowledge of danger signs and complications during the PNC period

**Figure 2:**
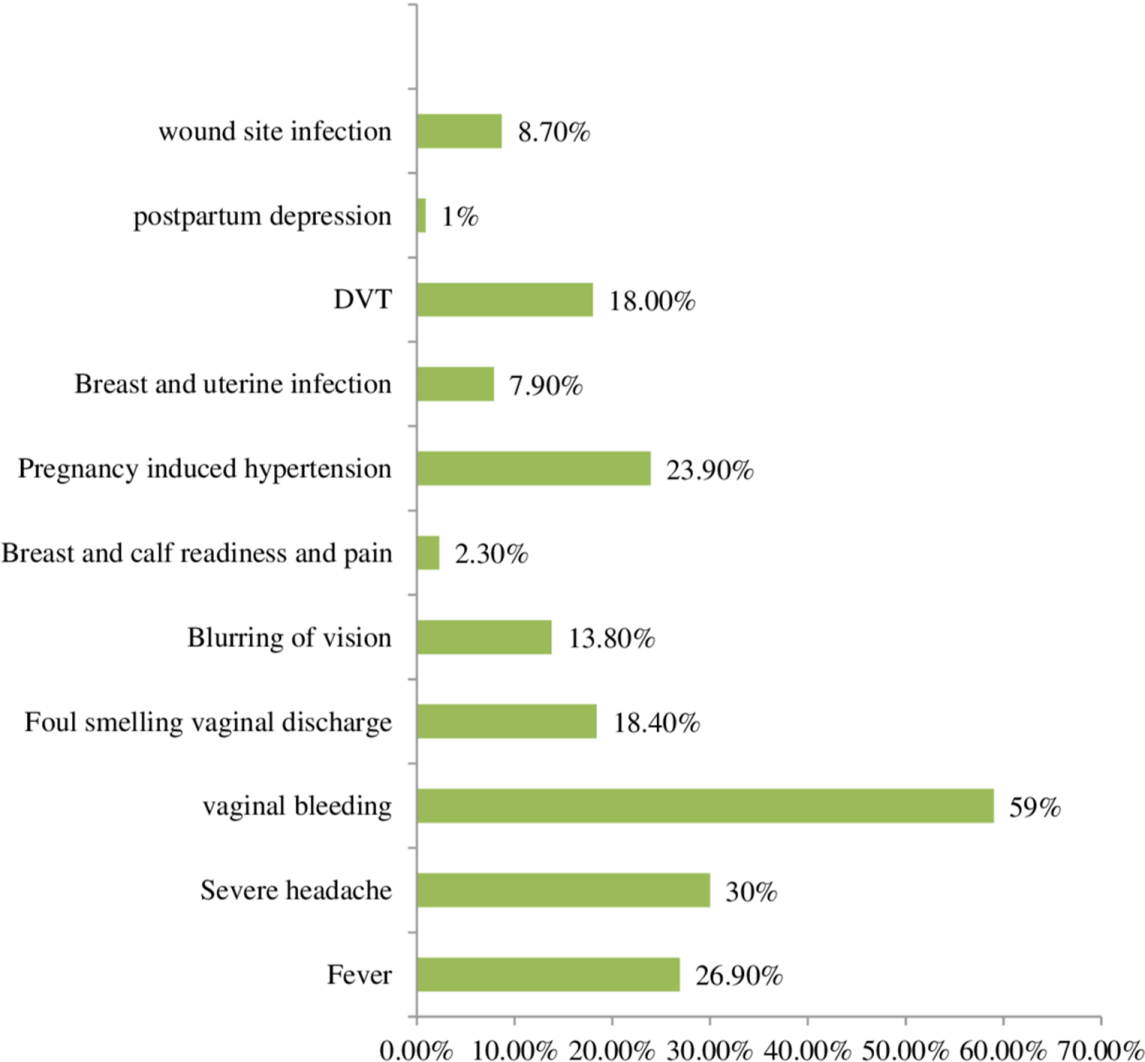
Male partner's knowledge of danger signs and complications during the PNC period in Wolaita Sodo, southern Ethiopia, 2023 (*N* = 610).

A total of 57.9% of all the participants had good knowledge of maternal danger signs or complications (i.e., mentioned three or more danger signs and complications) that could happen during the postpartum period. The study participants most frequently reported vaginal bleeding among the danger signs (59.0%), whereas depression (1%) was the least frequently reported sign, as shown in Figure 2.

### 3.4 Attitude toward PNC services

Of the 610 respondents, 257 (42.1%) had a favorable attitude toward PNC services. Of these 135 (22.1%) and 228 (37.4%) strongly disagreed and disagreed with the idea that it was a waste of time for male partners to accompany their wives during PNC visits, respectively. However, 181 (29.7%) strongly agreed and 140 (23.0%) agreed with the statement that mothers should follow PNC follow-up within 42 days (Table 2).

**Table 2:** Attitude of male partners toward PNC services in Wolaita Sodo, southern Ethiopia, 2023 (*N* = 610).

Variable	Frequency (%)				
	Strongly agree	Agree	Neutral	Disagree	Strongly disagree
It is a waste of time for male partners to accompany their wives to PNC visits	18 (3.0)	60 (9.8)	169 (27.7)	228 (37.4)	135 (22.1)
Male partners should give emotional, financial, and physical support to their wives during the PNC period	147 (24.1)	331 (54.3)	89 (14.6)	25 (4.1)	18 (3.0)
Male partners must focus on job responsibilities during the postnatal period rather than be involved in caring for their wives and children	12 (2.0)	61 (10)	116 (19)	228 (37.4)	193 (31.6)
PNC services should only be left to women alone	18 (3.0)	60 (9.8)	121 (19.8)	215 (35.2)	196 (32.1)
Visiting health institutions during the postnatal period is important for mothers and their children	192 (31.5)	223 (36.6)	121 (19.8)	39 (6.4)	35 (5.7)
Recommend other males to be involved in PNC services	48 (7.9)	74 (12.1)	352 (57.7)	105 (17.2)	31 (5.1)
Mothers should follow PNC follow-up within 42 days	181 (29.7)	140 (23.0)	208 (34.1)	35 (5.7)	46 (7.5)

### 3.5 Health service-related characteristics

In total, 96.1% of the study participants had transport access to the PNC clinic and the wives of 93.9% gave birth in the health institution. A total of 243 respondents (39.8%) reported being able to access health facilities (PNC clinics) within 30 min. Regarding the mode of delivery, 99 (16.2%) of the respondents' wives delivered by cesarean delivery. Regarding the question of whether health professionals provide a good welcome to men during maternal health service utilization, 124 respondents (20.3%) reported receiving a warm welcome from health professionals when they accompanied their partner to the PNC clinic for services. Furthermore, 137 (22.4%) respondents reported that there was privacy when the service was provided. Finally, 20.7% of the respondents received the service within 1 h.

### 3.6 Male partner involvement in PNC service utilization

**Figure 3:**
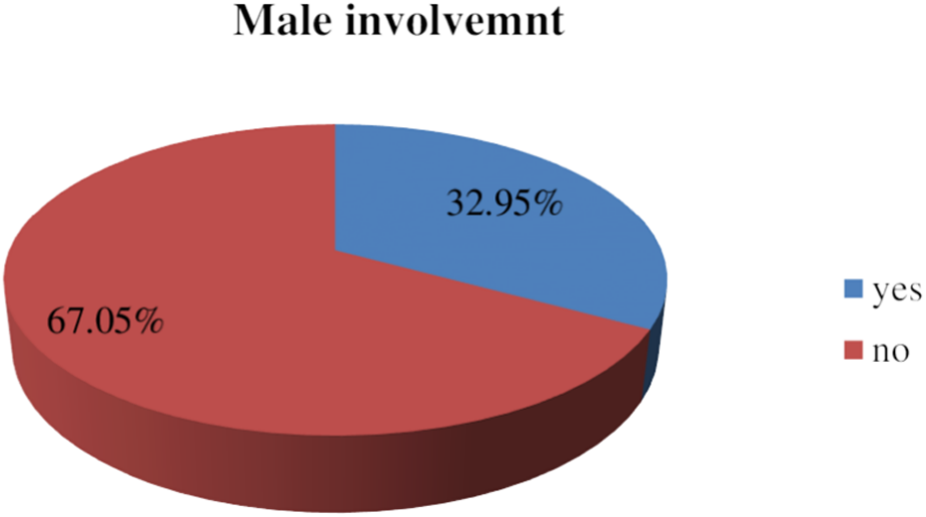
Prevalence of male partners” involvement in postnatal care services in Wolaita Sodo, southern Ethiopia, 2023 (*N* = 610).

Overall, 201 (32.95%) of all the men included in the study were involved in postnatal care service utilization with a 95% CI (29.2–36.8). Of these, 168 (27.5%) respondents accompanied their partner to the health facility for PNC service utilization (Figure 3).

Out of the total, 378 (62.0%) male partners reported that they had family planning discussions with their partner. When asked if they provided financial support to their partner for PNC service utilization, 449 respondents (73.6%) confirmed that they did. In addition, 26.6% discussed PNC services and complications during the postpartum period with their healthcare providers (Table 3).

**Table 3:** Male partners’ involvement in PNC service utilization in Wolaita Sodo, southern Ethiopia, 2023 (*N* = 610).

Components	Category	Frequency	Percentage
Discusses PNC services with their partner	Yes	298	48.9
	No	312	51.1
Shared decision-making regarding PNC with wife	Yes	259	42.5
	No	351	57.5
Accompanies partner to the healthcare facility	Yes	168	27.5
	No	442	72.5
Discusses family planning with their partner	Yes	378	62.0
	No	232	38.0
Discusses PNC services and complications during the postpartum period with wife’s healthcare provider	Yes	162	26.6
	No	448	73.4
Provides physical support to his partner during the postnatal period	Yes	264	43.3
	No	346	56.7
Provides emotional support to their partner (encouragement) for PNC service utilization	Yes	349	57.2
	No	261	42.8
Provides financial support to their partner for PNC service utilization	Yes	449	73.6
	No	161	26.4
Helps with domestic tasks	Yes	421	69.0
	No	189	31.0
Looks after children	Yes	519	85.1
	No	91	14.9

### 3.7 Factors associated with male partner involvement in PNC service utilization

In the bivariable analysis, age, educational status, maternal educational status, number of children, knowledge of PNC, knowledge of danger signs and complications, attitude, distance, and mode of delivery were significantly associated with male partner involvement in PNC service utilization at a *P*-value <0.25. Of the variables found to be significant in bivariable analysis, wife’s educational status (college or above) (AOR: 2.95, 95% CI: 1.76–4.94), good knowledge of PNC services (AOR: 3.2, 95% CI: 1.93–5.3), good knowledge of danger signs and complications that occur during the postpartum period (AOR: 4.5, 95% CI: 2.39–8.48), a favorable attitude toward PNC services (AOR: 4.02, 95% CI: 2.50–6.45), health facility (PNC clinic) access within 30 min (AOR: 1.91, 95% CI = 1.15–3.17), and wife delivered by cesarean delivery [AOR: 2.5, 95% CI: 1.39–4.60] were found to be associated with male partner involvement in PNC service utilization (Table 4).

**Table 4:** Bivariable and multivariable logistic regression of factors associated with male partner involvement in PNC service utilization in Wolaita Sodo, southern Ethiopia, 2023 (*N* = 610).

Variable	Male involvement		COR (95% CI)	AOR (95% CI)	*P*-value
	Yes	No			
Age (years)					
18–25	14 (25.5%)	41 (74.5%)	1.2 (0.86–1.69)	0.59 (0.35–1.2)	0.580
26–35	142 (40.5%)	209 (59.5%)	2.4 (1.924–3.37)	1.17 (0.7–1.96)	0.817
>35	45 (22.1%)	159 (77.9%)	1	1	
Male partners’ education					
No formal education	30 (21.1%)	112 (78.9%)	1	1	
Primary (1–8)	44 (22.0%)	156 (78.0%)	1.05 (0.75–1.47)	0.51 (0.3–1.05)	0.540
Secondary (9–12)	48 (36.4%)	84 (63.6%)	2.133 (1.519–2.997)	1.04 (0.6–1.7)	0.898
Collage and above	79 (58.1%)	57 (41.9%)	5.174 (3.685–7.269)	2.5 (0.98–4.2)	0.917
Wives’ education					
No formal education	25 (19.1%)	106 (80.9%)	1	1	
Primary (1–8)	50 (23.1%)	166 (76.9%)	1.277 (0.892–1.760)	0.61 (0.36–1.02)	0.924
Secondary (9–12)	36 (32.4%)	75 (67.6%)	2.035 (1.422–2.806)	0.97 (0.58–1.63)	0.107
College and above	90 (59.2%)	62 (40.8%)	6.155 (4.301–8.485)	2.9 (1.7–4.9)	0.005*
Number of children					
1–2	108 (38.3%)	174 (61.7%)	1.56 (1.11–2.2)	0.76 (0.46–1.28)	0.312
≥3	93 (28.4%)	235 (71.6%)	1	1	
Average monthly income					
<4,500	70 (26.9%)	190 (73.1%)	1	1	
≥4,500	131 (37.4%)	219 (62.6%)	1.6 (1.14–2.3)	1.39 (0.8–2.3)	0.200
Knowledge of PNC					
Good	155 (57.0%)	117 (43.0%)	8.4 (5.6–12.4)	3.1 (1.9–5.3)	0.000**
Poor	46 (13.6%)	292 (86.4%)	1	1	
Knowledge of danger signs					
Good	179 (50.7%)	174 (49.3%)	10.9 (6.7–17.8)	4.5 (2.4–8.4)	0.000**
Poor	22 (8.6%)	235 (91.4%)	1	1	
Attitude					
Favorable	136 (52.9%)	121 (47.1%)	4.98 (3.46–7.16)	4.01 (2.5–6.44)	0.000**
Unfavorable	65 (18.4%)	288 (81.6%)	1	1	
Distance (min)					
≤30	110 (45.3%)	133 (54.7%)	2.50 (1.77–3.55)	1.91 (1.15–3.17)	0.013***
>30	91 (24.8%)	276 (75.2%)	1	1	
Mode of delivery					
SVD	141 (27.6%)	370 (72.4%)	1	1	
CS	60 (60.6%)	39 (39.4%)	4.03 (2.58–6.31)	2.5 (1.4–4.6)	0.002*

AOR, adjusted odd ratio; COR, crude odds ratio, 1 reference; SVD, spontaneous vaginal delivery; CS, cesarean section.

* Significant at *P*-value < 0.01; **Significant at *P*- value < 0.001; ***Significant at *P*-value < 0.05.

## 4 Discussion

This community-based cross-sectional study aimed to assess the extent of male partner involvement in PNC service utilization and to identify key factors influencing this involvement, including socio-demographic, individual, and health facility factors in Wolaita Sodo, southern Ethiopia. The prevalence of male partner involvement in PNC service utilization in this study was found to be 32.95.0% (95% CI: 29.2–36.8). This is comparable to similar studies conducted in Nepal and Ghana which reported 33.8% (11) and 31.76% (20), respectively. However, the prevalence observed in this study is higher than that in other districts of Ghana and the Motta District in Ethiopia, where the rates were 20% (21) and 20.8% (14), respectively. The observed discrepancy could be attributed to several factors, including differences in geographic locations, socio-economic conditions, or health system characteristics. For example, socio-demographic factors, such as education levels and cultural norms, may play a role in shaping male partner involvement in maternal health services. In addition, variations in the health systems of the study settings, such as the availability of PNC services and male engagement strategies, could explain these differences.

However, studies conducted in Tanzania, Nigeria, and Addis Ababa, Ethiopia, reported higher male involvement rates in PNC services, with figures of 59.3% (22), 87.7% (12), and 54.7% (14), respectively. These differences may be due to methodological variations in measuring male involvement and contextual factors such as differences in local health policies, public health campaigns, or the role of community health workers. These higher figures may also reflect more robust male engagement programs or cultural norms that are more accepting of male participation in maternal health.

The study found that male partners whose wives had completed college or higher education were 2.95 times more likely to be involved in PNC services compared to those whose wives had no formal education. This finding is consistent with a previous study in Myanmar (23). The possible explanation might be that educated women are more likely to have decision-making autonomy, better access to health-related information, and financial resources, all of which may encourage their partners to participate in postnatal care (24). Furthermore, educated women often have better communication with their husbands, which facilitates joint decision-making regarding maternal and child health (25).

The current study exhibited that male partners who had good knowledge of PNC services were 3.2 times more likely to be involved in PNC service utilization as compared to those who had poor knowledge of the service, which was supported by studies conducted in India (26), Bangladesh (27) and Tanzania (16). Understanding the importance of PNC services is a crucial determinant of male involvement. Knowledgeable men are more likely to recognize the importance of PNC visits for the health of their wives and children, thus encouraging them to accompany their partners to health facilities. Increasing male awareness about maternal health risks and benefits may therefore serve as a key intervention to improve male participation in PNC services. Male partners who had good knowledge of danger signs and complications that occur during the postpartum period were 4.5 times more likely to be involved in PNC service utilization than their counterparts. This aligns with findings from India (28), which suggest that awareness of postpartum danger signs significantly influences male participation in maternal health. When male partners are informed about potential complications, they are more likely to accompany their wives to healthcare facilities to ensure timely medical intervention, thus enhancing the overall health outcomes for mothers and newborns (29). In addition, awareness of danger signs increases readiness to deal with obstetric difficulties (17).

This study showed that male partners who had a favorable attitude toward PNC service were 4.02 times more likely to be involved in PNC service utilization than their counterparts. This finding was consistent with previous studies (30, 31) and highlights the importance of attitudes in shaping health behaviors. A positive attitude toward PNC services may motivate men to provide logistical and financial support, such as transportation and accompanying their wives to healthcare visits. Interventions aimed at changing male attitudes through targeted health education campaigns may thus play a critical role in increasing male involvement in maternal healthcare (32).

In terms of health facility-related factors, the study found that male partners who had access to a PNC clinic within 30 min of their home were 1.91 times more likely to be involved in PNC services than those living farther away. This finding is consistent with studies in Iran (32) and Ghana (21), which demonstrate that proximity to healthcare facilities is a significant determinant of healthcare utilization. Men living closer to healthcare facilities may be more likely to accompany their wives due to reduced travel time and transportation costs. Conversely, those living farther away may face challenges such as high transportation costs, time constraints, or logistical barriers that prevent them from attending PNC visits with their spouses. Finally, the study found that male partners whose wives delivered via cesarean section were 2.5 times more likely to participate in PNC services than those whose wives delivered vaginally. This finding was supported by a study conducted in Gondar (33), which suggests that women who undergo cesarean sections are at a higher risk of complications and may require more care and attention from their husbands during the postpartum period. Male involvement in the care of women recovering from cesarean deliveries may, therefore, be a response to the heightened awareness of the need for maternal care and support in such cases (33).

The findings of this study have important implications for policy and practice in improving male partner involvement in PNC services. Interventions aimed at increasing male engagement in maternal health should focus on enhancing male awareness and knowledge of PNC services, danger signs, and the importance of postpartum care. Educational campaigns and community-based programs should also target attitudes and perceptions about male involvement, with an emphasis on overcoming cultural and social barriers that may hinder participation. In addition, improving access to healthcare facilities and addressing logistical barriers, such as transportation costs and travel time, may increase male participation, particularly in rural areas. Policymakers should consider integrating male engagement strategies into maternal health programs to foster more inclusive and holistic approaches to maternal and child healthcare.

## 5 Limitations of the study

This study faces the limitations commonly associated with cross-sectional research, as it cannot establish a direct cause-and-effect relationship. In addition, the absence of rural participants limits the generalizability of the findings to rural settings. Furthermore, the study did not include mothers who delivered outside of wedlock, which could provide important insights into male involvement in postnatal care. Finally, the study did not explore cultural factors that may influence the perspectives of the participants, which may be critical for understanding the full scope of male partner involvement in PNC.

## 6 Conclusion

This study found that 32.95% of the male partners were involved in PNC service utilization. Although this rate is higher than that reported in other regions, it remains relatively modest compared to studies that have documented higher levels of male participation.

This study identified several key factors associated with increased male involvement, including the educational status of their wives, awareness of PNC services, knowledge of postpartum danger signs, a positive attitude toward PNC, proximity to PNC clinics, and the mode of delivery (cesarean section).

Given these results, it is crucial to raise awareness about PNC services and postpartum danger signs through targeted health education campaigns aimed at men. Efforts should also focus on promoting positive attitudes toward PNC services to encourage greater male involvement. Additionally, comprehensive studies employing various designs are needed to explore cultural factors not addressed in this research, including the inclusion of mothers who gave birth outside of wedlock and the underlying barriers and challenges that impede male participation.

## Data Availability

The raw data supporting the conclusions of this article will be made available by the authors, without undue reservation.
